# Reduced Body Weight and Increased Energy Expenditure in Transgenic Mice Over-Expressing Soluble Leptin Receptor

**DOI:** 10.1371/journal.pone.0011669

**Published:** 2010-07-20

**Authors:** Phing-How Lou, Guoqing Yang, Lu Huang, Yunxia Cui, Tiffany Pourbahrami, George K. Radda, Cai Li, Weiping Han

**Affiliations:** 1 Laboratory of Metabolic Medicine, Singapore Bioimaging Consortium, Agency for Science, Technology and Research (A*STAR), Singapore, Singapore; 2 Department of Physiology, Touchstone Center for Diabetes Research, The University of Texas Southwestern Medical Center, Dallas, Texas, United States of America; 3 Department of Biochemistry, Yong Loo Lin School of Medicine, National University of Singapore, Singapore, Singapore; University of Hong Kong, China

## Abstract

**Background:**

Soluble leptin receptor (OBRe), one of several leptin receptor isoforms, is the only bona fide leptin binding protein in plasma. Our earlier studies demonstrated that OBRe modulates leptin levels in circulation. Both clinical and *in vitro* studies have shown that OBRe expression is inversely correlated to body weight and leptin levels. However, it is not clear whether OBRe plays an active role, either in collaboration with leptin or independently, in the maintenance of body weight.

**Methodology/Principal Findings:**

To investigate the function of OBRe in the regulation of energy homeostasis, we generated transgenic mice that express OBRe under the control of human serum amyloid P (hSAP) component gene promoter. The transgene led to approximately doubling of OBRe in circulation in the transgenic mice than in wild type control mice. Transgenic mice exhibited lower body weight at 4 weeks of age, and slower rate of weight gain when compared with control mice. Furthermore, transgenic mice had lower body fat content. Indirect calorimetry revealed that transgenic mice had reduced food intake, increased basal metabolic rate, and increased lipid oxidation, which could account for the differences in body weight and body fat content. Transgenic mice also showed higher total circulating leptin, with the majority of it being in the bound form, while the amount of free leptin is comparable between transgenic and control mice.

**Conclusions:**

These results are consistent with the role of OBRe as a leptin binding protein in regulating leptin's bioavailability and activity.

## Introduction

Obesity, a result of disruption in energy homeostasis, is increasingly becoming a prevalent global pandemic. Numerous studies have shown the importance of leptin in the regulation of energy homeostasis [1], [2], [3], [4], [5]. By acting on the satiety center in the hypothalamus, leptin reduces appetite and increases energy expenditure [6]. In humans and rodents, mutations of the gene encoding leptin lead to severe early-onset obesity, which can be corrected by leptin treatment [4], [5], [7], [8], [9].

Leptin achieves its control on metabolic processes through its interaction with the leptin receptor (OBR) [10], a member of the class I cytokine family of receptors [11]. There are at least five leptin receptor isoforms, OBRa to OBRe, which may be generated by alternative splicing [12] or ectodomain shedding [13]. All the leptin receptors share a common N-terminal extracellular ligand-binding sequence, but differ considerably with variable length of C-terminal regions. Only the full-length receptor, OBRb has been shown to transmit leptin signal through its C-terminal tyrosine residues and associated proteins, and is responsible for most, if not all of the leptin's regulatory function in energy homeostasis, as the mutation that specifically ablates OBRb expression causes a phenotype that is indistinguishable from that in animals without leptin [10], [12], [14].

The functions and significance of the shorter isoforms of leptin receptors are not known or fully understood. OBRa through OBRd may mediate the degradation or additional intracellular action of leptin by facilitating its endocytosis into cells [15], although OBRa and OBRc were earlier proposed to transport leptin into brain across the blood-brain barrier (BBB) given their abundant expression in the choroid plexus of the BBB [16]. In contrast, OBRe, also known as soluble leptin receptor (SLR) due to its lack of the transmembrane and cytoplasmic regions, was shown to inhibit leptin transport into the brain [17].

OBRe is the only bona fide leptin-binding protein in circulation, with similar leptin-binding affinity as OBRb [18]. The close correlation of OBRe and leptin levels in energy homeostasis has been demonstrated in several clinical studies: OBRe level is inversely correlated with obesity and leptin levels [19], [20], [21]; OBRe expression is increased in response to weight loss, fasting and food restriction [21], [22]; and a higher proportion of OBRe-bound leptin is present in circulation in the lean than the obese individuals [21], [23]. Moreover, the amount of OBRe in circulation also seems to be influenced by leptin [19]. However, it is not clear whether OBRe plays an active role, either in collaboration with leptin or independently, in the maintenance of body weight. Previous studies on OBRe were limited to cellular level [17] or adenovirus-mediated transient over-expression in mouse [24]. To directly evaluate the long-term effects of OBRe on metabolism and leptin action *in vivo*, we generated a transgenic mouse line with specific over-expression of OBRe in the liver.

## Materials and Methods

### Animal welfare

All mice used in this study were bred and housed in our animal facility. They were maintained at 25±1°C on a 12 h/12 h light/dark cycle (7:00–19:00 h), and allowed free access to water and rodent chow (15% kcal from fat; Harland Tekland, WI). All experiments involving animals were reviewed and approved by the Institutional Animal Care and Use Committee of the University of Texas Southwestern Medical Center in Dallas (0873-04-07-1) and Agency for Science, Technology and Research (A*STAR) Biomedical Science Institutes in Singapore (050129 and 080351).

### Generation of OBRe over-expressing mice (hSAP-OBRe)

**Figure 1:**
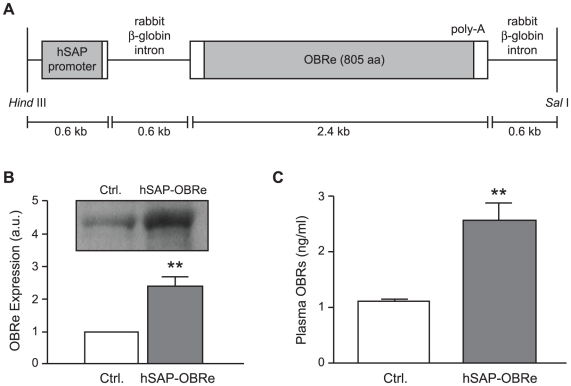
Structure of the OBRe transgene and its expression in hSAP-OBRe mice. (A) Full-length mouse OBRe cDNA and poly-adenylation signal, flanked by rabbit β-globin introns, were placed downstream of the hSAP component promoter. The size of each fragment is indicated in the diagram. *Hind* III and *Sal* I were used to linearize the transgene fragment before the microinjection procedure. (B) A representative Western blot (OBRe) of blood plasma from a 20- to 24-week old male hSAP-OBRe transgenic mouse and its littermate control. Below the Western blot is the relative densitometric quantification of OBRe from 4 independent pairs of hSAP-OBRe transgenic and control mouse plasma samples. (C) Quantification of soluble leptin receptors from plasma samples of male hSAP-OBRe transgenic (N = 13) and control (N = 11) mice using ELISA. Data are presented as means ± SEM. **, P<0.01. a.u.: arbitrary units.

The *hSAP-OBRe* transgene, containing the mouse *OBRe* cDNA under the control of the human serum amyloid P (hSAP) promoter, was constructed by placing the 2.4 kb *OBRe* cDNA and a poly-adenylation signal between rabbit β-globin introns (Figure 1A). The backbone vector (without the mouse *OBRe* cDNA insert) was a kind gift from J. Miyazaki (Osaka University), whose laboratory developed the promoter construct and generated transgenic mice over-expressing the p40 subunit of IL-12 [25]. The full length *OBRe* cDNA was amplified by PCR and cloned into pSG-2's EcoRI site by blunt ligation. The 4.2 kb transgene fragment was excised from its vector by restriction digestion with HindIII and SalI, and purified for microinjection. Transgenic mice were identified by PCR of tail DNA using the following primers: 5′-GTG GTA AAG ACT TGA GGT GAA C-3′ and 5′-GAA TTA TGA CTC TAA GGT CCA TC-3′. The founder mice were generated on a hybrid background, and were backcrossed to the C57Bl6 background for 7 generations.

### Body weight and composition measurements

Age-matched littermates of hSAP-OBRe transgenic and control mice were weighed weekly after weaning, and their body composition was measured at age of 23–24 weeks by using an EchoMRI-100 (Echo Medical Systems) essentially as previously described [26].

### Plasma collection

Blood from tail bleeding was collected in EDTA-coated Eppendorf tubes (final concentration 5 mM), and tubes were centrifuged at 8,000 g for 5 min at 4°C. Plasma was collected and used for leptin and leptin receptor analysis, or stored at −80°C for future use.

### Western blotting

Five micro-litres of plasma was separated on an SDS/5% polyacrylamide gel and transferred to PVDF membrane. The membrane was then probed for OBRe with 0.2 µg/ml mouse anti-leptin receptor antibody (sc-8391, Santa Cruz Biotechnology), followed by HRP-conjugated goat anti-mouse antibody.

### Quantification of plasma OBRe

OBRe concentration in plasma was measured by Mouse Leptin R DuoSet ELISA assay (R&D Systems) with a detection limit of 15.6 pg/ml. The kit measures total leptin receptor, either bound or free. Each concentration was determined from an average of duplicate assays.

### Gel-filtration chromatography for leptin separation

Bound and free fractions of leptin were separated by gel filtration chromatography at 4°C. Each plasma sample (200 µl) was incubated with 2 µl of murine recombinant ^125^I-leptin (100,000 cpm, Perkin Elmer) overnight at 4°C. Of the 200 µl of prepared plasma mixture, only 100 µl was loaded and size-fractionated using a Superdex 200 (GE) column on an ÄKTA Purifier chromatography system with 25 mM phosphate-buffered saline (137 mM NaCl, 2.7 mM KCl, 8 mM Na_2_HPO_4_, 1.46 mM KH_2_PO_4_, pH, 7.2) as the elution buffer. The amount of radioactivity from each fraction was measured on a Perkin Elmer Wallac Wizard 1470 Gamma Counter. In a typical elution profile, the first peak represents the bound leptin while the last peak represents the free leptin. The proportions of bound and free ^125^I-leptin were determined by analyzing the areas under the curve of both peaks of the chromatographic profile. Absolute concentrations of bound and free leptin were calculated by multiplying the percentage of bound and free leptin, respectively, by the total leptin concentration and dividing by 100. Total leptin concentrations were measured by ELISA assay (LINCO Research). Each concentration was averaged from duplicate assays.

### Oxymax/Comprehensive Lab Animal Monitoring System (CLAMS)

Oxymax/CLAMS (Columbus Instruments) was used to quantitate individual mice on their oxygen consumption (VO_2_), carbon dioxide production (VCO_2_), activity, and feed intake. Mice (16 week-old) were individually housed in chambers maintained at 24±1°C, and given free access to chow and water. All the measurements were taken every 15 minutes for 6 days after the mice were acclimatized for 1 day. Basal metabolic rate (BMR) was determined by averaging lowest plateau region of oxygen consumption curve corresponding to resting periods. The respiratory exchange ratio (RER) was calculated as the ratio between VCO_2_ and VO_2_. All data collected were averaged from 6 days' monitoring.

### Statistical analysis

Data are presented as means ± SEM. Comparisons of data were made by using two-tailed Student's t-test. The significant limit was set at P<0.05.

## Results

### Generation of hSAP-OBRe mice

To test the effects of increased circulating OBRe on energy homeostasis, we generated transgenic mice to achieve consistent and sustained OBRe over-expression. Expression of the transgene was under the hSAP promoter (Figure 1A), which has been used previously to drive high-level expression specifically in livers of transgenic mouse models [27], [28]. To determine whether the *hSAP-OBRe* transgene resulted in a higher level of OBRe in the circulatory system, we compared OBRe levels in the plasma of the transgenic and control mice by using Western blot analysis (Figure 1B) and ELISA assay (Figure 1C). In both methods, plasma OBRe level in the hSAP-OBRe mice was approximately twice of that in the control mice. These results confirmed the higher level of OBRe expression in the hSAP-OBRe mice.

### Decreased body weight and body fat in hSAP-OBRe mice

**Figure 2:**
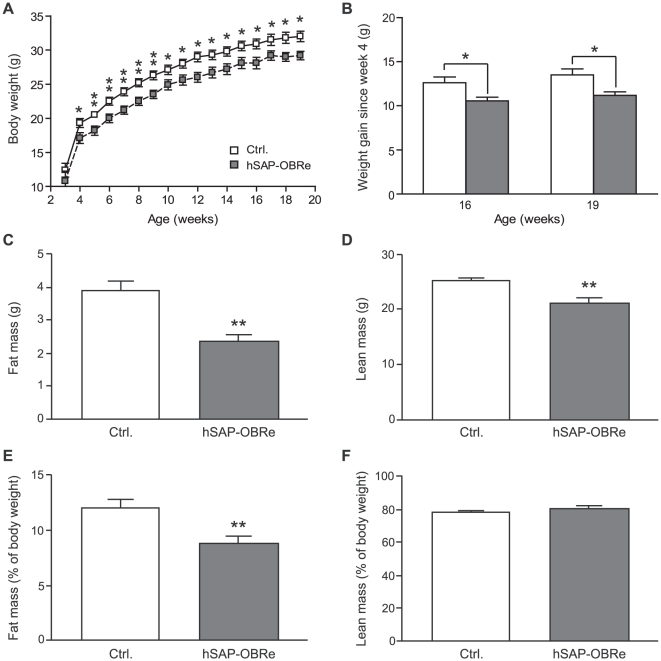
Reduced body weight and body fat mass in hSAP-OBRe transgenic mice. (A) Body weight of male hSAP-OBRe transgenic and control mice given a standard rodent chow diet with measurements taken at three (N = 4 mice per genotype) or four weeks (N = 9–13 mice per genotype) after birth. (B) Amount of weight gained by 16- or 19-week old hSAP-OBRe and control mice since the fourth postnatal week; data taken from (A). (C, D) Absolute amounts of body fat (C) and lean mass (D) in male hSAP-OBRe transgenic and control mice at age 24 weeks (N = 10 for each genotype). (E, F) Proportion of body fat (E) and lean mass (F) in hSAP-OBRe transgenic and control mice at 24 weeks (N = 10 for each genotype), which was calculated as the percentage of their respective body weights. Data are presented as means ± SEM. *, P<0.05; **, P<0.01.

An earlier study using adenovirus-mediated gene transfer showed transient OBRe over-expression enhanced leptin's effect on body weight and food intake in *ob/ob* mice [24]. The availability of hSAP-OBRe mice, with consistent and sustained increase in OBRe level, made it possible to determine the long-term effect of OBRe on energy homeostasis. We first measured the body weight of male transgenic and control mice weekly for a period of 16 weeks after weaning (Figure 2A). At 4 weeks old, hSAP-OBRe mice weighed significantly less than their wild type littermates, and this body weight difference persisted throughout the monitoring period (Figure 2A). To compare the rate of weight gain between the two genotypes, we calculated the amount of weight difference since week 4 in hSAP-OBRe and control mice. At week 16 and 19 (pre- and post-metabolic chamber analysis, respectively), hSAP-OBRe mice gained significantly less weight than the control mice, thus suggesting a slower rate of weight gain in the transgenic mice (Figure 2B). A similar trend was also observed in the F6 hybrid background mice that were continuously fed either a high-fat diet (60% kcal fat, HFD) or a low-fat diet (10% kcal fat, LFD) (Figure S1A–B). The growth curves under different diets showed similar patterns in the two genotypes, with a larger body weight gain over the first 5–7 weeks after birth, followed by a progressively slower weight gain in subsequent weeks.

At the end of the 16-week body weight monitoring, we analyzed the total body fat and lean mass for both groups by MRI (magnetic resonance imaging). hSAP-OBRe mice had significantly lower total fat and lean mass compared with their control (Figure 2C, 2D). As the transgenic mice weighed less than the control group, we normalized the fat and lean mass to body weight to evaluate whether the difference was merely due to the body weight difference. After normalization to body weight, the proportion of body mass in fat in the hSAP-OBRe mice was still lower than their wild type littermates (Figure 2E), while the proportion of lean body mass was not different (Figure 2F). These data suggest that increased OBRe may have a specific effect in reducing fat accumulation, in addition to a general effect in preventing body weight gain.

### Reduced food intake and increased energy expenditure in hSAP-OBRe mice

**Figure 3:**
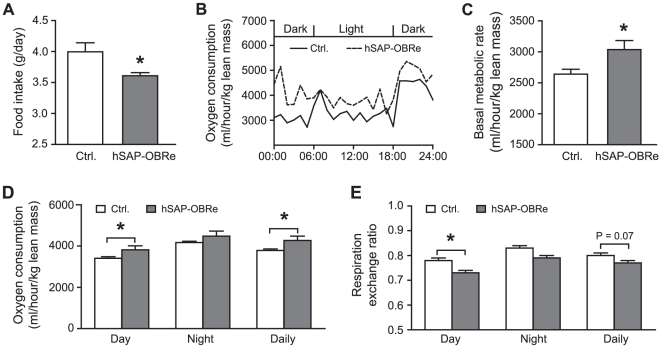
hSAP-OBRe mice had lower food intake and higher basal metabolic rate. (A) Daily food consumption of hSAP-OBRe transgenic and control mice over a 6-day period for 16- to 17-week old mice. Data are presented as an average of 6 days' measurement. (B) Circadian rhythm of oxygen consumption in hSAP-OBRe transgenic (dashed line) and control (solid line) mice. Data shown are the representative oxygen consumption profiles of a hSAP-OBRe transgenic and control mouse over a 24-hour monitoring period. (C) BMR (ml/hour/kg lean mass) for hSAP-OBRe transgenic and control mice. (D) Rate of daily oxygen consumption for hSAP-OBRe transgenic and control mice during the day, night and the whole-day periods. (E) RER of hSAP-OBRe transgenic and control mice during the day, night, and the whole-day period. All data, except in (B), are presented as means ± SEM from the same population of male mice, with each genotype comprising 10 animals. *, P<0.05.

To understand the cause of the lower body weight and body fat in transgenic mice, we examined their daily food intake and energy expenditure together with their littermate control mice by using the Oxymax/CLAMS system. The tests were done over a 6-day period after 1 day of acclimatization. All mice had free access to food and water, and were subjected to the same dark-light cycle during the tests. Transgenic mice consumed ∼11% less food than their control littermates (Figure 3A), and showed higher oxygen consumption during the day, and the whole-day periods (Figure 3B and 3D). Furthermore, BMR, as measured by basal oxygen consumption, was significantly higher in hSAP-OBRe mice (Figure 3C). We also tested locomotor activity to examine whether higher oxygen consumption in transgenic mice could be accounted for by increased physical activity, however, there was no difference between hSAP-OBRe transgenic and control mice (70054±13409 vs. 69562±5173, N = 10 in each genotype). As the hSAP-OBRe transgenic mice had lower body fat, we tested whether the transgenic mice showed increased use of fat as energy source by quantifying RER. hSAP-OBRe exhibited a significant reduction in RER especially during the day (Figure 3E), suggesting that the transgenic mice used a higher proportion of fat in their energy production than their control mice.

### Increased total but unchanged free leptin in hSAP-OBRe mice

**Figure 4:**
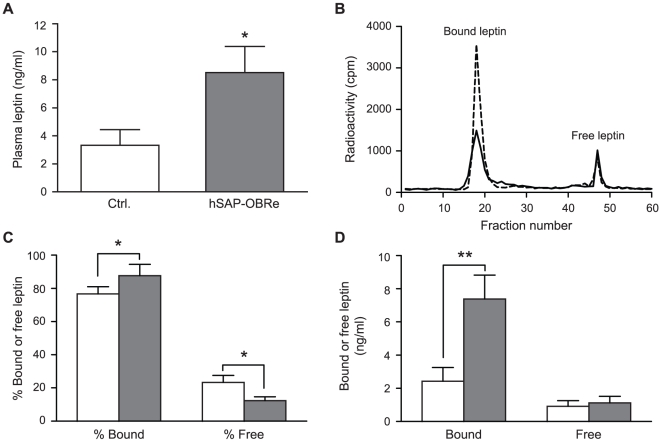
Increased total, but unchanged free plasma leptin in hSAP-OBRe transgenic mice. (A) Concentration of total plasma leptin from hSAP-OBRe transgenic and control mice. (B) An example of an elution profile (from FPLC) of plasma from hSAP-OBRe transgenic (dashed line) and control (solid line) mouse. The first peak represents bound leptin, whereas the last peak represents free leptin. (C) Proportion of bound and free plasma leptin based on the areas under the curve of both peaks of the chromatographic profile. (D) Absolute concentrations of bound and free leptin derived from the total plasma leptin in (A) based on the proportion of each leptin form (C). All data shown, except (B), are compiled from the same population of 20- to 24-week old male mice (N = 12–13 animals per genotype), and are given as means ± SEM. *, P<0.05; **, P<0.01.

OBRe is a major leptin-binding protein in blood [18], and higher level of OBRe in circulation may modulate free leptin concentration by altering the distribution of free and bound leptin, and consequently affect leptin's action in the maintenance of energy homeostasis. To test this hypothesis, we first measured total plasma leptin in transgenic and control mice by ELISA, and found that hSAP-OBRe mice had significantly higher leptin levels (Figure 4A).

We next determined the distribution of free and bound leptin in plasma of hSAP-OBRe transgenic and littermate control mice by gel filtration chromatography. When ^125^I-leptin was incubated with plasma and fractionated, two peaks of radioactivity were observed (Figure 4B). Typical elution profiles of ^125^I-leptin-treated plasma of hSAP-OBRe transgenic and control mice are shown in Figure 4B. The first peak represents leptin bound to high molecular mass protein, presumably OBRe, while the second peak represents free monomeric leptin (Figure 4B). Transgenic mice showed apparently higher peak level of radioactivity for bound leptin than the control mice (Figure 4B). To estimate the proportion of bound and free leptin from the elution profiles, we calculated the area under the curve of each peak, and found hSAP-OBRe mice had a higher proportion of leptin circulating in bound form and a smaller proportion of circulating free leptin when compared with the wild type mice (Figure 4C). When the percentages of the leptin proportions were calculated into absolute concentrations, a higher level of circulating leptin was present as the bound form in the hSAP-OBRe mice, while the amount of free leptin was similar between the transgenic and control mice (Figure 4D).

## Discussion

Here, we investigated the long-term metabolic impact of soluble leptin receptor (OBRe) over-expression *in vivo* by analysing the OBRe over-expressing hSAP-OBRe mice. Because OBRe may be generated by either alternative splicing or ectodomain shedding of membrane containing OBRs [13], [29], [30], it is not possible to study the function of OBRe *in vivo* by knocking out OBRe specifically at the protein level without affecting the other OBRs. As such, our transgenic mice represent a valuable model to investigate the *in vivo* function of OBRe and to study its interaction with leptin and other relevant proteins in energy homeostasis, especially considering that the level of OBRe over-expression in these transgenic mice was just over two-fold compared with wild type levels (Figure 1B and 1C), an increase that is within physiological range. Too high an expression level of a protein sometimes causes artefacts that mask the real consequence of physiological increases of the protein *in vivo* [31], [32].

Compared with wild type mice, hSAP-OBRe mice had lower body weights (Figure 2A), and lower fat mass (Figure 2C and 2E). These phenotypes could be due to reduced food intake (Figure 3A) and increased metabolic rate (Figure 3B–D), both of which may be the result of enhanced leptin action on energy regulation [2], [33], [34], [35]. There are at least three possible scenarios as how OBRe functions in leptin-regulated energy homeostasis:

First, OBRe may regulate leptin availability and thus its biological activity [36]. Leptin circulates in both free and protein-bound form, and OBRe is a major leptin-binding protein in circulation [11], [23], [37]. At 16 kDa, leptin would be more rapidly cleared or metabolized as a monomer than when leptin is in a much bigger protein complex, such as when bound to OBRe [19], which may delay leptin clearance, and thus raise blood leptin levels [24]. However, bound leptin is inactive because OBRb-binding site on leptin is occupied by OBRe, which shares the same leptin binding motif with OBRb [18]. Although we cannot rule out that leptin-bound OBRe may play a role in energy homeostasis, it is more likely that OBRe-bound leptin serves as a reservoir to maintain a constant pool of readily available leptin [24], [36].

Second, OBRe may regulate leptin transport into the cerebrospinal fluid (CSF), and thus the amount of leptin at target sites in the hypothalamus. Free leptin is transported into the CSF by a high affinity but low capacity transport system in contrast to bound leptin, whose transport is not easily saturated [38]. Therefore, there may be an increase in the total amount of leptin in CSF in hSAP-OBRe mice. However, it is not practical to test this hypothesis, because it is technically very difficult to extract CSF in sufficient amount from mice for a reliable quantification of leptin levels.

Third, OBRe may perform a regulatory role in energy homeostasis independent of free leptin. Although the proportion of free leptin was lower in hSAP-OBRe transgenic mice than in control mice, it is worth noting that the absolute levels of free leptin were similar between the two groups (Figure 4C and 4D). When this is considered, together with the fact that the hSAP-OBRe mice had lower body weight and body fat with reduced food intake and increased energy expenditure, it is tempting to suggest that OBRe may play an active role in regulating body weight and energy homeostasis independent of free leptin. This hypothesis may be tested in future studies that isolate the contributions of leptin and OBRe, such as introducing the hSAP-OBRe transgene into the leptin-deficient *ob/ob* mice.

In summary, our results demonstrate that OBRe is an active component, either in collaboration with leptin as part of an OBRe-leptin complex or independent of free leptin, in the regulation of energy homeostasis. The finding extends the current understanding of the functions of leptin receptor isoforms, and suggests OBRe as a potential target in the management of obesity and related disorders.

## Supporting Information

Figure S1 Reduced body weight in hSAP-OBRe hybrid background mice. (A) Body weight measurements of male hSAP-OBRe hybrid background and control mice given HFD (N = 14 per genotype) or LFD (N = 12–15 per genotype) for 14 weeks. Arrow indicates the start of the diet regime. (B) Amount of weight gained by 18-week old hSAP-OBRe and control mice since the third postnatal week; data taken from (A). Asterisks in red indicate significance within the HFD groups, while those in black indicate significance within the LFD groups. Data are presented as means ± SEM. *, P<0.05; **, P<0.01.
(0.47 MB EPS)

## References

[1] Campfield LA, Smith FJ, Guisez Y, Devos R, Burn P (1995). Recombinant mouse OB protein: evidence for a peripheral signal linking adiposity and central neural networks.. Science.

[2] Halaas JL, Gajiwala KS, Maffei M, Cohen SL, Chait BT (1995). Weight-reducing effects of the plasma protein encoded by the obese gene.. Science.

[3] Friedman JM (2002). The function of leptin in nutrition, weight, and physiology.. Nutr Rev.

[4] Zhang Y, Proenca R, Maffei M, Barone M, Leopold L (1994). Positional cloning of the mouse obese gene and its human homologue.. Nature.

[5] Montague CT, Farooqi IS, Whitehead JP, Soos MA, Rau H (1997). Congenital leptin deficiency is associated with severe early-onset obesity in humans.. Nature.

[6] Friedman JM, Halaas JL (1998). Leptin and the regulation of body weight in mammals.. Nature.

[7] Chehab FF, Lim ME, Lu R (1996). Correction of the sterility defect in homozygous obese female mice by treatment with the human recombinant leptin.. Nat Genet.

[8] Farooqi IS, Jebb SA, Langmack G, Lawrence E, Cheetham CH (1999). Effects of recombinant leptin therapy in a child with congenital leptin deficiency.. N Engl J Med.

[9] Licinio J, Caglayan S, Ozata M, Yildiz BO, de Miranda PB (2004). Phenotypic effects of leptin replacement on morbid obesity, diabetes mellitus, hypogonadism, and behavior in leptin-deficient adults.. Proc Natl Acad Sci U S A.

[10] Myers MG, Cowley MA, Munzberg H (2008). Mechanisms of leptin action and leptin resistance.. Annu Rev Physiol.

[11] Tartaglia LA, Dembski M, Weng X, Deng N, Culpepper J (1995). Identification and expression cloning of a leptin receptor, OB-R.. Cell.

[12] Lee GH, Proenca R, Montez JM, Carroll KM, Darvishzadeh JG (1996). Abnormal splicing of the leptin receptor in diabetic mice.. Nature.

[13] Ge H, Huang L, Pourbahrami T, Li C (2002). Generation of soluble leptin receptor by ectodomain shedding of membrane-spanning receptors in vitro and in vivo.. J Biol Chem.

[14] Chen H, Charlat O, Tartaglia LA, Woolf EA, Weng X (1996). Evidence that the diabetes gene encodes the leptin receptor: identification of a mutation in the leptin receptor gene in db/db mice.. Cell.

[15] Tu H, Pan W, Feucht L, Kastin AJ (2007). Convergent trafficking pattern of leptin after endocytosis mediated by ObRa-ObRd.. J Cell Physiol.

[16] Hileman SM, Pierroz DD, Masuzaki H, Bjorbaek C, El-Haschimi K (2002). Characterizaton of short isoforms of the leptin receptor in rat cerebral microvessels and of brain uptake of leptin in mouse models of obesity.. Endocrinology.

[17] Tu H, Kastin AJ, Hsuchou H, Pan W (2008). Soluble receptor inhibits leptin transport.. J Cell Physiol.

[18] Liu C, Liu XJ, Barry G, Ling N, Maki RA (1997). Expression and characterization of a putative high affinity human soluble leptin receptor.. Endocrinology.

[19] Chan JL, Bluher S, Yiannakouris N, Suchard MA, Kratzsch J (2002). Regulation of circulating soluble leptin receptor levels by gender, adiposity, sex steroids, and leptin: observational and interventional studies in humans.. Diabetes.

[20] Ogier V, Ziegler O, Mejean L, Nicolas JP, Stricker-Krongrad A (2002). Obesity is associated with decreasing levels of the circulating soluble leptin receptor in humans.. Int J Obes Relat Metab Disord.

[21] Shimizu H, Shimomura K, Negishi M, Masunaga M, Uehara Y (2002). Circulating concentrations of soluble leptin receptor: influence of menstrual cycle and diet therapy.. Nutrition.

[22] Cohen SE, Kokkotou E, Biddinger SB, Kondo T, Gebhardt R (2007). High circulating leptin receptors with normal leptin sensitivity in liver-specific insulin receptor knock-out (LIRKO) mice.. J Biol Chem.

[23] Sinha MK, Opentanova I, Ohannesian JP, Kolaczynski JW, Heiman ML (1996). Evidence of free and bound leptin in human circulation. Studies in lean and obese subjects and during short-term fasting.. J Clin Invest.

[24] Huang L, Wang Z, Li C (2001). Modulation of circulating leptin levels by its soluble receptor.. J Biol Chem.

[25] Yoshimoto T, Wang CR, Yoneto T, Waki S, Sunaga S (1998). Reduced T helper 1 responses in IL-12 p40 transgenic mice.. J Immunol.

[26] Gustavsson N, Lao Y, Maximov A, Chuang JC, Kostromina E (2008). Impaired insulin secretion and glucose intolerance in synaptotagmin-7 null mutant mice.. Proc Natl Acad Sci U S A.

[27] Matsuda J, Suzuki M, Nozaki C, Shinya N, Tashiro K (1998). Transgenic mouse expressing a full-length hepatitis C virus cDNA.. Jpn J Cancer Res.

[28] Zhao X, Araki K, Miyazaki J, Yamamura K (1992). Developmental and liver-specific expression directed by the serum amyloid P component promoter in transgenic mice.. J Biochem.

[29] Li C, Ioffe E, Fidahusein N, Connolly E, Friedman JM (1998). Absence of soluble leptin receptor in plasma from dbPas/dbPas and other db/db mice.. J Biol Chem.

[30] Maamra M, Bidlingmaier M, Postel-Vinay MC, Wu Z, Strasburger CJ (2001). Generation of human soluble leptin receptor by proteolytic cleavage of membrane-anchored receptors.. Endocrinology.

[31] Harper JA, Stuart JA, Jekabsons MB, Roussel D, Brindle KM (2002). Artifactual uncoupling by uncoupling protein 3 in yeast mitochondria at the concentrations found in mouse and rat skeletal-muscle mitochondria.. Biochem J.

[32] Stuart JA, Harper JA, Brindle KM, Jekabsons MB, Brand MD (2001). Physiological levels of mammalian uncoupling protein 2 do not uncouple yeast mitochondria.. J Biol Chem.

[33] Halaas JL, Boozer C, Blair-West J, Fidahusein N, Denton DA (1997). Physiological response to long-term peripheral and central leptin infusion in lean and obese mice.. Proc Natl Acad Sci U S A.

[34] Scarpace PJ, Matheny M (1998). Leptin induction of UCP1 gene expression is dependent on sympathetic innervation.. Am J Physiol.

[35] Doring H, Schwarzer K, Nuesslein-Hildesheim B, Schmidt I (1998). Leptin selectively increases energy expenditure of food-restricted lean mice.. Int J Obes Relat Metab Disord.

[36] Gavrilova O, Barr V, Marcus-Samuels B, Reitman M (1997). Hyperleptinemia of pregnancy associated with the appearance of a circulating form of the leptin receptor.. J Biol Chem.

[37] Lewandowski K, Horn R, O'Callaghan CJ, Dunlop D, Medley GF (1999). Free leptin, bound leptin, and soluble leptin receptor in normal and diabetic pregnancies.. J Clin Endocrinol Metab.

[38] Brabant G, Horn R, von zur Muhlen A, Mayr B, Wurster U (2000). Free and protein bound leptin are distinct and independently controlled factors in energy regulation.. Diabetologia.

